# The Neuropilin-1 Inhibitor, ATWLPPR Peptide, Prevents Experimental Diabetes-Induced Retinal Injury by Preserving Vascular Integrity and Decreasing Oxidative Stress

**DOI:** 10.1371/journal.pone.0142571

**Published:** 2015-11-10

**Authors:** Jun Wang, Shuaiwei Wang, Mengling Li, Dongdong Wu, Fang Liu, Ruisheng Yang, Shaoping Ji, Ailing Ji, Yanzhang Li

**Affiliations:** 1 Medical College of Henan University, Kaifeng, 475004, China; 2 The First Affiliated Hospital of Henan University, Kaifeng 475001, China; University of Florida, UNITED STATES

## Abstract

Neuropilin-1 (NRP-1) is a transmembrane glycoprotein. As a VEGF co-receptor, NRP1 significantly enhances VEGFR2 signaling and promotes vascular permeability and migration. The purpose of this study was to evaluate the effects of an NRP-1 inhibitor, ATWLPPR peptide, on the early stages of diabetic retinopathy. Eight-week-old male C57BL/6 mice were divided into three groups: a Normal group, a Diabetes (DB) ATWLPPR treatment group and a DB saline group. Electroretinography (ERG), fundus fluorescence angiography (FFA) and leukostasis were examined to evaluate the retinal injury induced by diabetes at the end of the fifth week after STZ injection. Occludin expression and extravasation of albumin were measured to determine the extent of vascular injury. The oxidative stress level and the levels of inflammation-associated proteins were also assayed. The results indicated that treatment with ATWLPPR prevents the abnormal condition of ERG (amplitudes of b-wave decreased and implicit time increased) and vascular injury (occludin degradation and increase in extravasated albumin). These effects were associated with a reduction in the oxidase stress level and the expression of VEGF, GFAP, and ICAM-1. We conclude that ATWLPPR, an NRP-1 inhibitor, may reduce the early retinal damage induced by diabetes by preserving vascular integrity and decreasing the oxidative stress level. Blockade of NRP-1 may be a new therapeutic strategy for the early stages of DR.

## Introduction

Diabetic retinopathy (DR) is one of the most common causes of blindness. The earliest and most significant change in DR is blood-retinal barrier (BRB) dysfunction. The pathological hallmarks of BRB dysfunction include loss of tight junction integrity, oxidative stress and inflammatory changes. The tight junctions of retinal capillary endothelial cells, covered with pericytes and Muller cells (glial cells), form the inner BRB, whose function is to remove toxic compounds and to prevent the free diffusion of substances between the blood and the retina [1]. BRB dysfunction is an important element in diabetes-induced retinal injury, which is associated with inflammatory and oxidative changes [2,3].

Vascular endothelial growth factor (VEGF) is an angiogenic factor and a vasopermeability factor, which is induced by conditions of hypoxia or high glucose [4,5]. The levels of VEGF in the vitreous fluid have been recognized as a marker of the severity of DR [5]. The main pathophysiologic effects of VEGF in diabetic retinopathy affect the endothelial tight junctions, increase vascular permeability, cause leukocyte aggregation in the microvasculature, through the activation of VEGFR2 signaling, and result in local cytokine production and increased inflammation [6]. Anti-VEGF therapy is currently an important clinical strategy for preventing DR [7].

VEGF receptor-2 (VEGFR2) is thought to play the most prominent role in angiogenesis and vascular permeability given that it is highly expressed on retinal endothelial cells. Once VEGFR2 achieves an activated state, Flk-1/KDR undergoes phosphorylation at several tyrosine residues, and VEGF signal cascades are initiated [8,9]. Neuropilin-1 (NRP-1) is a non-tyrosine kinase transmembrane glycoprotein that enhances the interaction between VEGF and KDR and amplifies the angiogenic effects of this signal transduction [10]. The peptide, ATWLPPR, was identified by screening a mutated phage library for affinity to an anti-VEGF165 monoclonal antibody [11]. ATWLPPR showed anti-angiogenic properties both in vivo and in vitro through its specific binding to NRP-1, the VEGF co-receptor, and its selective inhibition of NRP-1. A previous study demonstrated that ATWLPPR inhibits tumor angiogenesis and growth [11]. Recently, an investigation indicated that the inhibition of NRP-1 by ATWLPPR preserved vascular integrity and enhanced survival in a blood-brain barrier disruption model [12]. However, little is known about the effects of this peptide on retinal vascular injury, particularly the BRB disruption and vascular inflammation induced by DR. This study sought to investigate whether ATWLPPR prevents the experimental diabetes-induced retinal injury in the early stages and to explore the possible involved mechanisms involved in vascular integrity.

## Materials and Methods

### 2.1 Animal ethics statement

Eight-week-old male C57BL/6 mice were obtained from the Model Animal Research Center of Nanjing University. The mice were maintained in a 12-h light/dark cycle with a humidity of 60 ±5% and a temperature of 22 ± 4°C. The animal protocols were approved by the Committee of Medical Ethics and Welfare for Experimental Animals, Henan University School of Medicine (Approval Number: MEWEAHUM 2014–0001). All surgeries were performed under anesthesia (ketamine/xylazine), and all efforts were made to minimize suffering.

### 2.2 Experimental diabetic mouse model

The diabetic mouse model is created by the intraperitoneal administration of 75 mg/kg of streptozotocin (STZ) on alternate days for up to 3 injections. The blood glucose levels of ≥16 mmol/L within 1 week of STZ treatment accompanied by polyuria and glucosuria were determined to be type 1 diabetes mellitus. The experimental type 1 DB models can be seen in previous reports [2, 13–15]. STZ was freshly prepared in 100 mM citrate buffer (pH 4.5). Blood glucose levels were measured using the YUYUE blood glucose monitor (ShangHai yuyue, China).

### 2.3 ATWLPPR peptide treatment

Sixty mice were randomly separated into three groups: Normal, Diabetes + ATWLPPR and Diabetes + saline. For the ATWLPPR treatment group, ATWLPPR peptide (dissolved in normal saline) was administered subcutaneously once daily (under the neck skin) at a dose of 400 μg/kg (0.1 ml/10 g body weight) from one week after the induction of diabetes. For the Diabetes + saline group, an equal volume of saline was administered subcutaneously. The Normal group involved a blank control and only underwent manipulation and subcutaneous needle insertion without any treatment. The ATWLPPR dose was determined based on demonstrated effectiveness in previous reports [12, 16].

### 2.4 Electroretinography

Focal ERG recordings were performed at the end of the 5^th^ week after treatment. Thirty-six animals were randomly assigned to three groups (12/group). For preparation of the ERG analysis, the mice underwent a dark adaptation for 3 hours. Their pupils were dilated with the application of tropicamide eye drops. Anesthesia was induced by injection of 60 mg/kg ketamine and 7 mg/kg xylazine. Focal ERG recordings were collected using a Micron III focal ERG system (Phoenix Research labs, USA). The corneal electrode was connected to the lens mount of the focal ERG. Platinum cutaneous needle electrodes as a reference were inserted into the posterior limb skin. The body temperature was kept at 37°C using a small animal electric blanket. An LED green light stimulus was projected onto the retina as a circular area, which was 4 times the diameter of the optic disc. The intensity was 40,000cd-s/m^2^ and the duration was 30 ms. Seventeen stimulus traces were averaged to generate the final ERG traces. The amplitude and implicit time of the b-wave were measured. The b-wave amplitude was from the minimum of the a-wave to the maximum of the b-wave. The implicit time was from the onset of the stimulus to the maximum of the b-wave. When these animals were awake for 24 hours, they underwent fluorescence angiography observation.

### 2.5 Fluorescence angiography

After anesthesia with the intraperitoneal application of a mixture containing ketamine (80 mg/kg) and xylazine (10 mg/kg), the pupils were dilated with 1% tropicamide and the cornea was kept moist using 1% carboxymethylcellulose sodium eye drops. The mice were injected intraperitoneally with 10% fluorescein sodium (Alcon, USA) at a dose of 1.5 ml/kg. The assay of fundus fluorescein angiography (FFA) was performed using a Micro-III retinal image system (Phoenix Research Labs Inc., USA). The acquisition of fundus images was performed 5 mins after the injection of fluorescein sodium at a rate of 4FPS.

### 2.6 Leukostasis

Leukostasis was evaluated based on the quantification of leukocytes that were adherent to the wall of the retinal vessels as described previously [2], with some modifications. In the mice, deep anesthesia was induced. Then, the chest cavity was opened, followed by the insertion of a perfusion cannula (internal diameter, 0.2 mm) into the aorta, and cutting of the right atrium. The animals were perfused with 10 ml of PBS (10 ml/min) to wash out the blood. Subsequently, 6 ml of FITC-labeled concanavalin A lectin (40 μg/ml in PBS, Sigma, USA) was perfused into the aortas at the same velocity of flow. The adherent leukocytes and vascular endothelial cells were labeled with concanavalin A. Unbound concanavalin A was removed by the subsequent perfusion with PBS. Eyeballs from each group (4 or 5 animals) were enucleated and fixed with 4% paraformaldehyde for 2 hours. The retinas were dissected and flat mounted on glass slides. The number of adherent leukocytes per retina was counted under fluorescence microscopy at X200 magnification.

### 2.7 Extravasation of albumin from the retinal vasculature

Under deep anesthesia, the mice underwent a thoracotomy operation. The right atrium was cut, and a heparinized catheter was inserted into the left ventricle. The mice were perfused with PBS at 37°C for 5 min through the heart to wash out the blood cells and proteins. The whole retinas were separated from the eyeballs, and Western blotting was performed to determine the vascular leakage by quantifying the extravascular albumin in the retinas. The retinas from 3 or 4 animals per group were collected for this experiment.

### 2.8 Western blot analysis

Retinas were excised quickly from the eyeballs, and then the retinal pigmented epithelium was carefully cleaned. Isolated retinas were placed into liquid nitrogen and transferred to a -80°C refrigerator. To extract the total protein, frozen retina specimens were homogenized in RIPA buffer (Beyotime Biotech, China). The protein concentration was measured using BCA protein assay kits (Beyotime Biotech). Each group included 3–4 animals (6–8 retinas); 30 μg of protein per sample was separated using 10% sodium dodecyl sulfate polyacrylamide gel electrophoresis and transferred onto nitrocellulose membranes (Millipore). The membranes were stained with Ponceau S solution to confirm protein loading. After washing, the membranes were blocked in 5% milk or 3% BSA in TBST (Tris-buffered saline with 0.5% Tween 20) and incubated overnight at 4°C with antibodies against GFAP (Proteintech, 1:2000), ICAM-1 (Boster, 1:400), VEGF (Boster, 1:400), albumin (Proteintech, 1:1000), and occludin (Santa Cruz, 1:400), followed by horseradish peroxidase-linked secondary antibodies (Boster, 1:5000). For the loading control, a β-actin antibody (CST, 1:5000) was used. Protein bands were visualized using ECL reagent (Solarbio, Beijing, China), followed by exposure on X-OMAT film (Kodak, Japan). The intensity of the protein bands was semi-quantitatively measured with image analysis software (Image J v2.1, USA), and β-actin was used for the calibration.

### 2.9 Immunohistochemistry

Paraffin-embedded slides were deparaffinized in xylene and rehydrated in decreasing concentrations of ethanol. After washing with 0.01 mol/l phosphate-buffered saline (PBS), antigen retrieval was performed using 0.01 M citrated buffer, pH 6.0, which was heated to 100°C in a microwave; the slides were then kept in the microwave for 10 min. Antigen retrieval was repeated two times. After blocking with 10% normal goat serum for 30 min, the sections were incubated with primary antibodies at 4°C overnight. After washing with PBS three times, the sections were incubated in appropriate concentrations of secondary antibodies for 1 hour at room temperature. After washing with PBS, the sections were imaged using fluorescence microscopy. Eyeballs from 3–4 animals per group were used for immunohistochemistry staining.

### 2.10 Measurement of MDA, SOD and ROS formation

Retinas from 4 mice in each group were isolated from the eyeballs. The samples were rinsed and homogenized using RIPA buffer (Beyotime, Shanghai, China). The supernatant homogenate was collected after centrifugation at 1600 g for 10 min at 4°C. SOD and MDA levels were measured using assay kits (Nanjing Jiancheng Bioengineering institute, Nanjing, China) according to the manufacturers’ instructions.

The levels of reactive oxygen species (ROS) in the retinal tissue sections were assayed using dihydroethidium (DHE). DHE, as a ROS fluorescent probe, penetrates through the cellular membrane and is oxidized into ethidium bromide by intracellular ROS. Ethidium bromide can bind to DNA in the nucleus and fluoresce red. Retinal cryosections from each group were incubated with DHE (5 μM for 40 min at room temperature). Images were obtained with a fluorescent microscope. To determine the specificity of the DHE reaction with ROS, we pre-incubated some sections with apocynin (1 mM) for 20 min, followed by incubation with DHE (5 μM for 40 min at room temperature). The relative fluorescent intensity was quantified using Image J software.

### 2.11 Statistical Analyses

All data are presented as the mean ± SD (or SE), and differences in means among the groups for non-repeated variables were compared using one-way ANOVA (Tukey test or Kruskal-Wallis ANOVA on ranks). *P* < 0.05 was considered to be significant. Sigma stat32 was used for the statistical analyses.

## Results

### 3.1 General description for body weight and blood glucose

**Fig 1:**
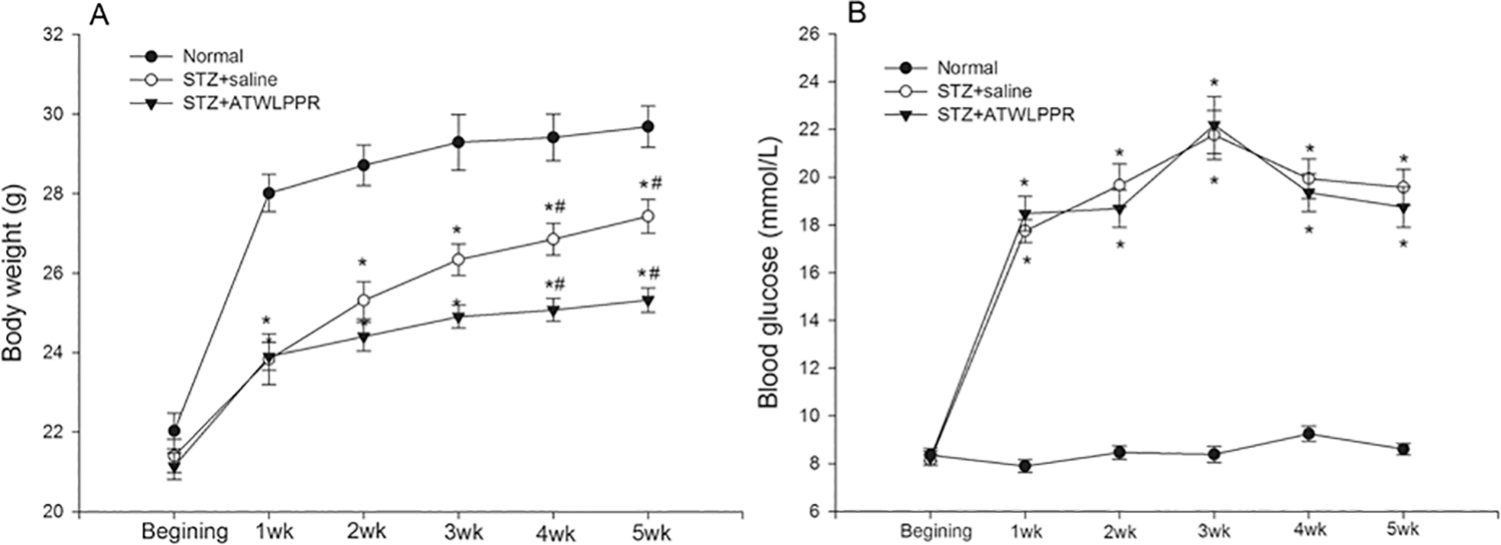
Changes in body weight and levels of blood glucose from the STZ injection to the end of the 5^th^ weekend. (A) body weight; (B) blood glucose (*P < 0.05, versus Normal; #*P* < 0.05 versus STZ+ATWLPPR).

As shown in Fig 1A, the body weight of the normal mice increased rapidly during the first week, and then remained slightly increased over the next few weeks. All diabetic animals (treated with ATWLPPR or with saline) showed a relatively slow increase in body weight, which appeared to be significantly lower than that of the normal mice from the end of STZ injected week (*P*<0.05). Unexpectedly, the peptide treatment led to a cessation of body weight growth compared with the DB saline animals, especially in the 4^th^ and 5^th^ week (Fig 1A). The blood glucose levels remained stable at a lower level in the normal group; however, high blood glucose levels continued throughout the five weeks in the STZ injected animals (Fig 1B, *P*<0.01, compared with that of the control group). There was no significant difference between the DB mice that were treated with and without ATWLPPR.

### 3.2 Electroretinography and fundus fluorescence angiography

**Fig 2:**
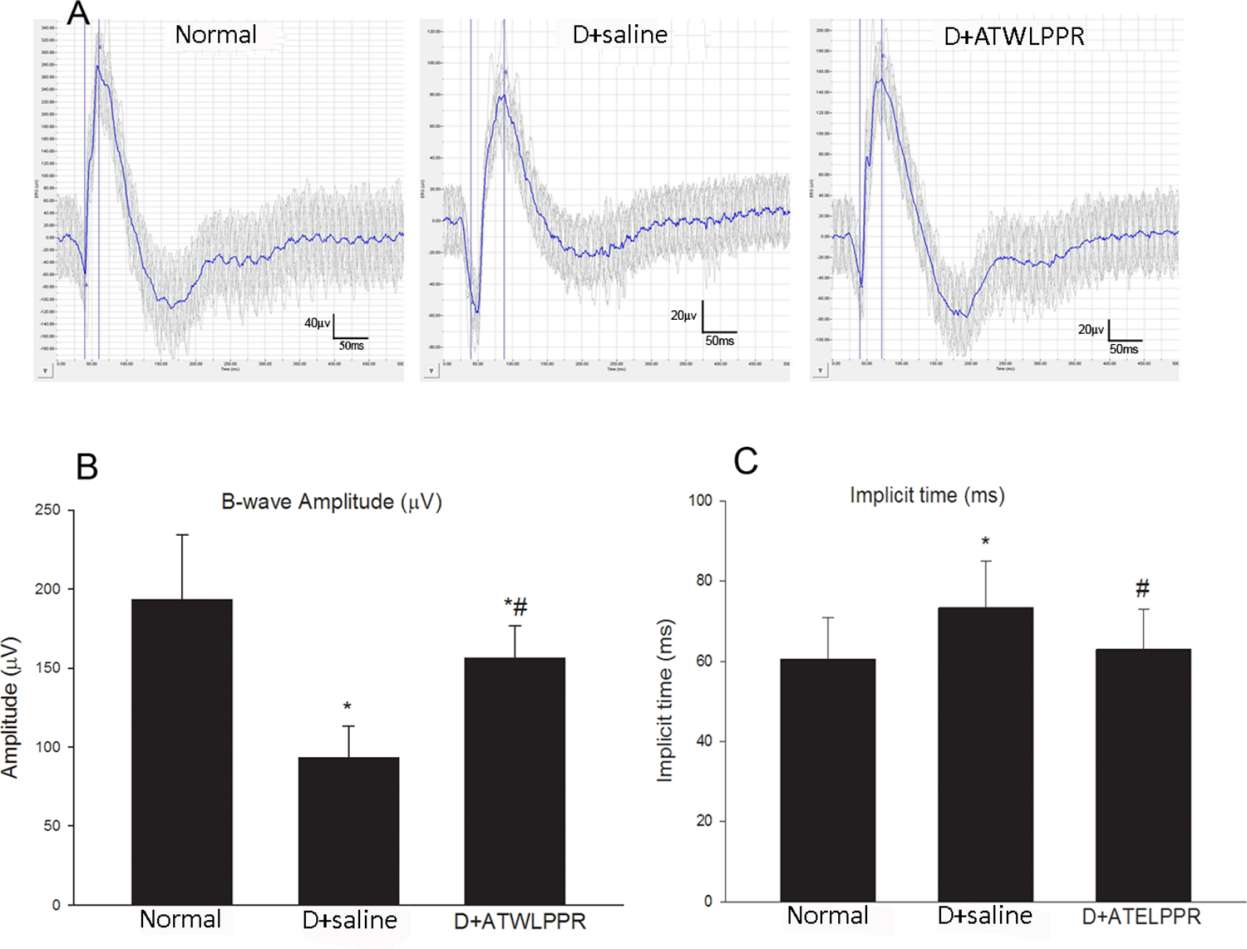
ERG responses in the Normal, D+saline and D+ATWLPPR groups. (A) Representative original recordings from the three groups; (B) Quantitative analysis of b-wave amplitudes (mV); (C) Quantitative analysis of b-wave implicit time (ms). *P < 0.05 versus the Normal group; #*P* < 0.05 versus the D+saline group. D, Diabetic.

Retinal function was evaluated using focal Phoenix Ganzfeld Electroretinography (ERG). Green light was chosen as the stimulatory spectrum. The alterations in implicit time and amplitude of the b-wave were observed in the ERG recordings. The b-wave was a reliable and sensitive marker for showing the function of multiple post-receptor neurons including bipolar, amacrine and ganglion cells. The results are shown in Fig 2. The b-wave amplitudes were significantly lower in the DB mice (treated with saline or ATWLPPR) compared with the normal animals (n = 12, each group; *P*<0.05). However, treatment with the neuropilin-1 inhibitor, ATWLPPR peptide, partially restored the b-wave amplitudes that were reduced in the DB saline group (Fig 2B). The ERG implicit time indicates the functional condition of the neural transduction pathway. The implicit time of b-wave was increased significantly in diabetic mice. However, in normal animals and in the group treated with the ATWLPPR peptide, the implicit time did not increase obviously at the end of the 5^th^ week (Fig 2C). Therefore, treatment with the neuropilin-1 inhibitor, ATWLPPR peptide, had protective effects against the abnormal ERG induced by diabetes.

**Fig 3:**
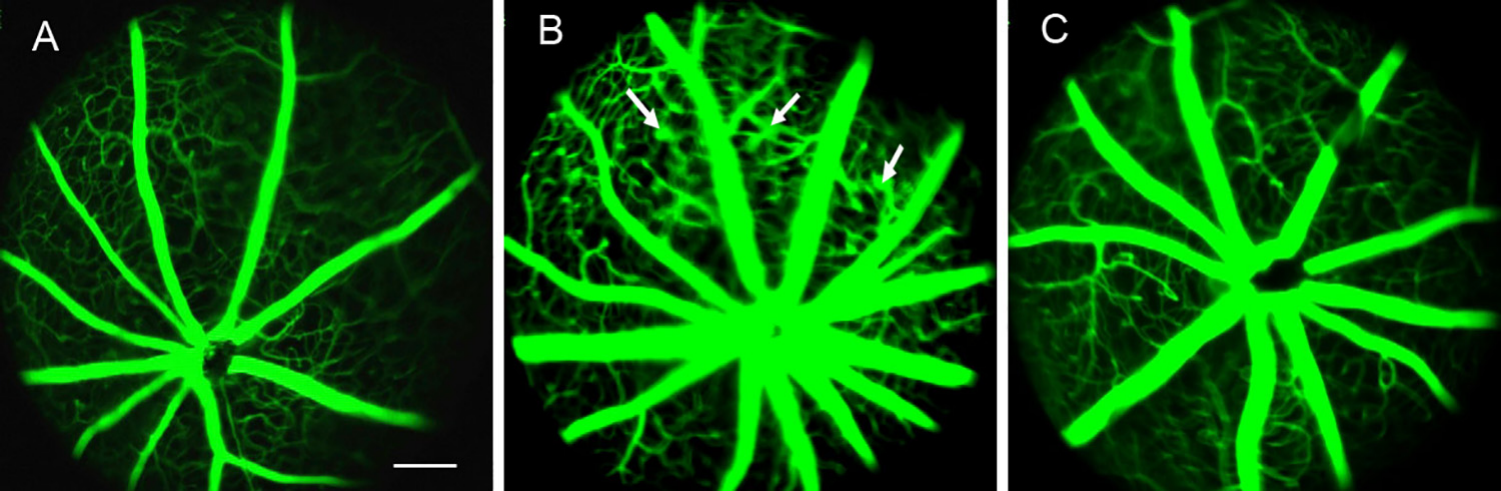
Representative FFA images. (A) Normal; (B) Diabetic; (C) Diabetic treated with ATWLPPR. Arrows: fluorescence leakage areas. Bar = 300 μm.

Fundus fluorescence angiography (FFA) was performed to observe the retinal vasculature in vivo. In diabetic ocular fundus examinations, some fluorescence leakage areas were observed, as shown in Fig 3. However, in normal mice and animals treated with ATWLPPR, this abnormal retinal vascular appearance was not observed (n = 12, each group).

### 3.3 Leukostasis

**Fig 4:**
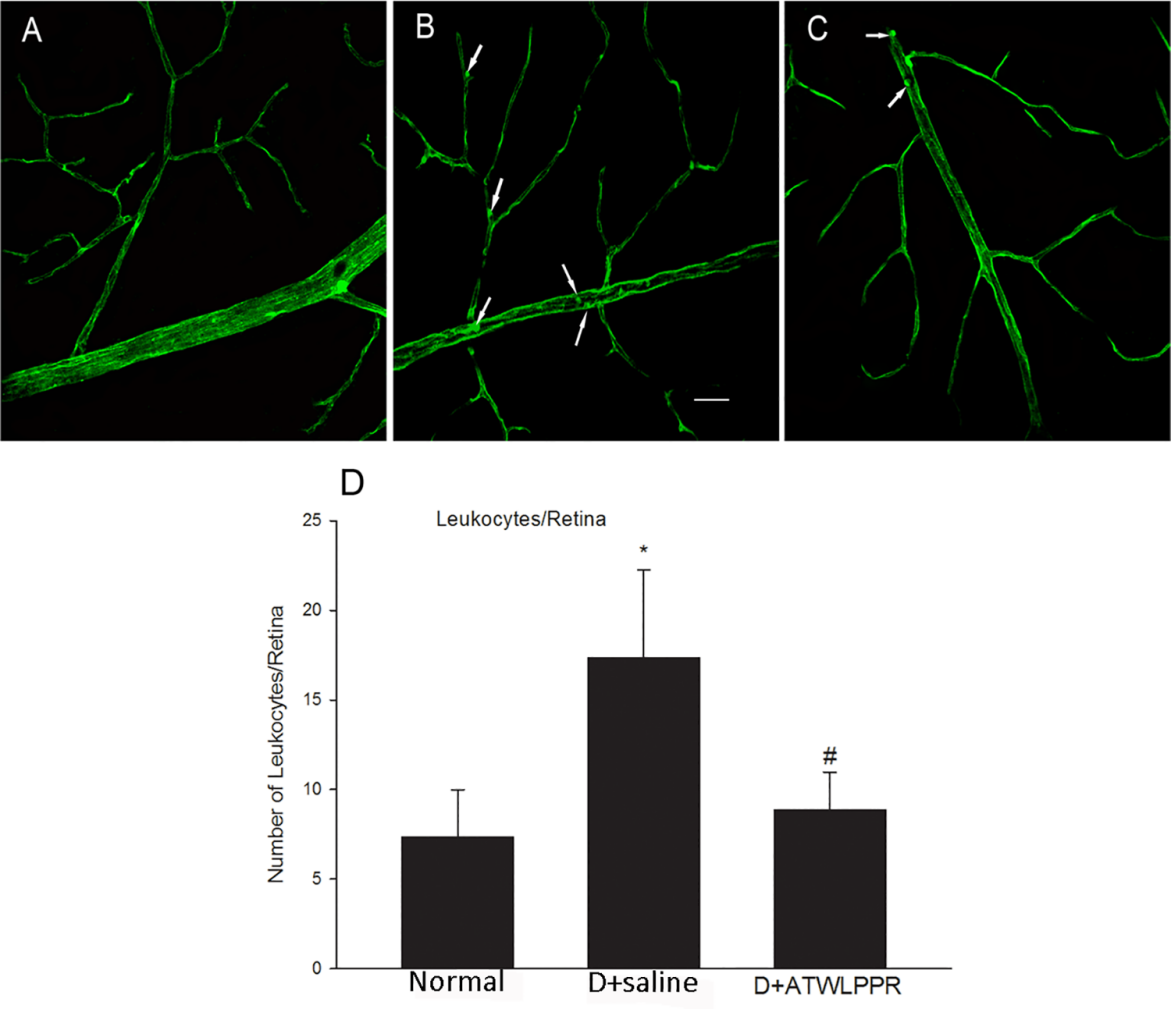
Treatment with ATWLPPR reduced the leukocyte attachment. Con A perfusion retinas show attached leukocytes (arrows) within the vasculature of diabetic retinas. Representative retina flat-mount images from (A) Normal, (B) D+saline and (C) D+ATWLPPR groups. (D) Quantitative analysis of leukocyte adhesion. Scale bar = 40 mm. *P < 0.05, versus the Normal group; #*P* < 0.05, versus the D+saline group. D, Diabetic.

To examine the effects of the ATWLPPR peptide on adherent diabetes-induced leucocytes and on the breakdown of the blood retinal barrier, vascular perfusion was performed to remove non-attached blood cells. The attached leukocytes were identified based on the perfusion of Con A and fluorescence microscopy imaging. Five weeks after the induction of diabetes with STZ, the number of adherent leukocytes was significantly higher than that in the non-diabetes controls. However, treatment with ATWLPPR significantly reduced the diabetes-induced leucocyte attachment (Fig 4).

### 3.4 Inhibition of NRP-1 prevents occludin degradation and extravasation of albumin

**Fig 5:**
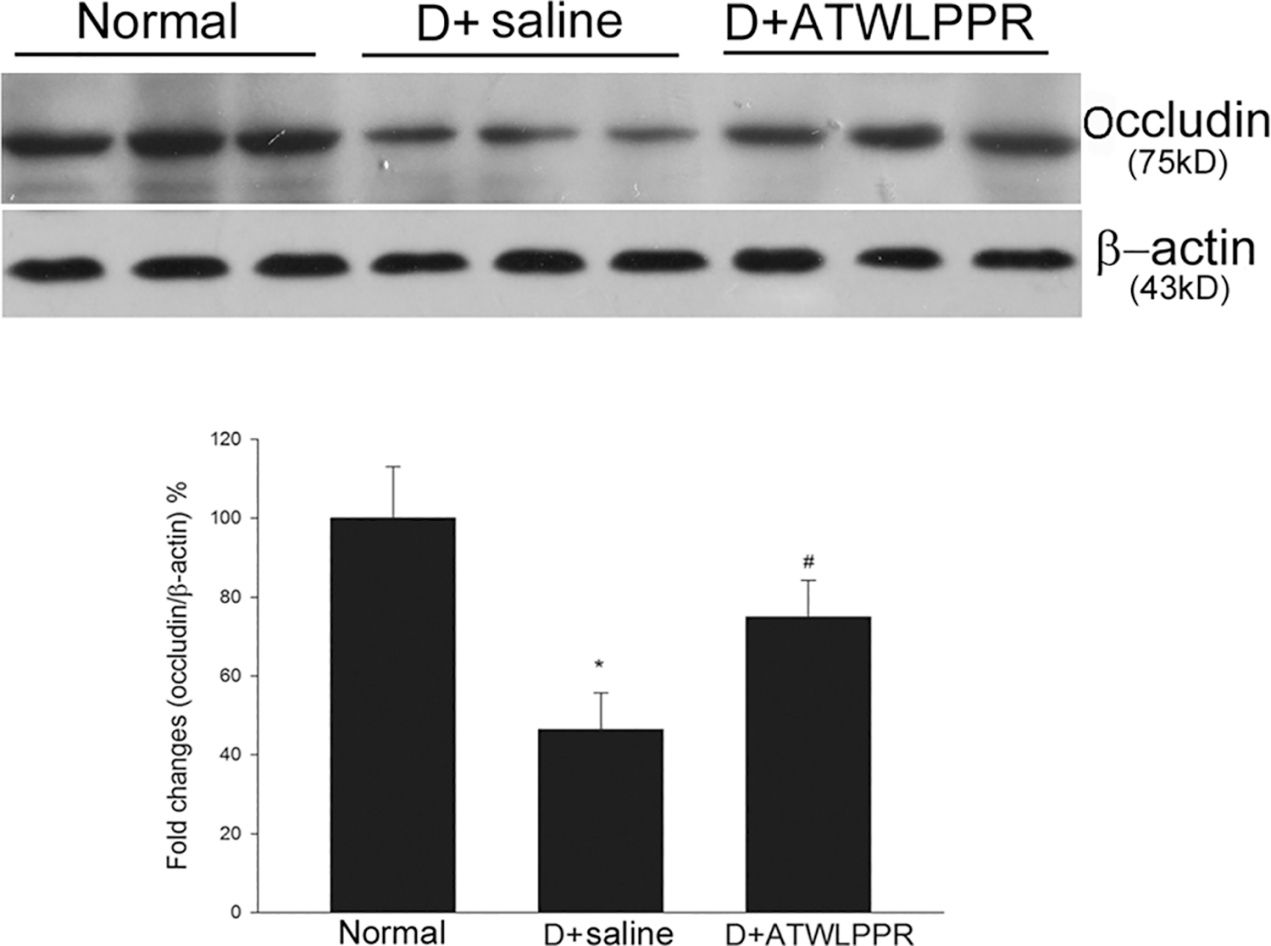
Diabetes-induced decrease in occludin was inhibited by treatment with ATWLPPR. *P < 0.05, versus the Normal group; #*P* < 0.05, versus the D+saline group. D, Diabetic.

**Fig 6:**
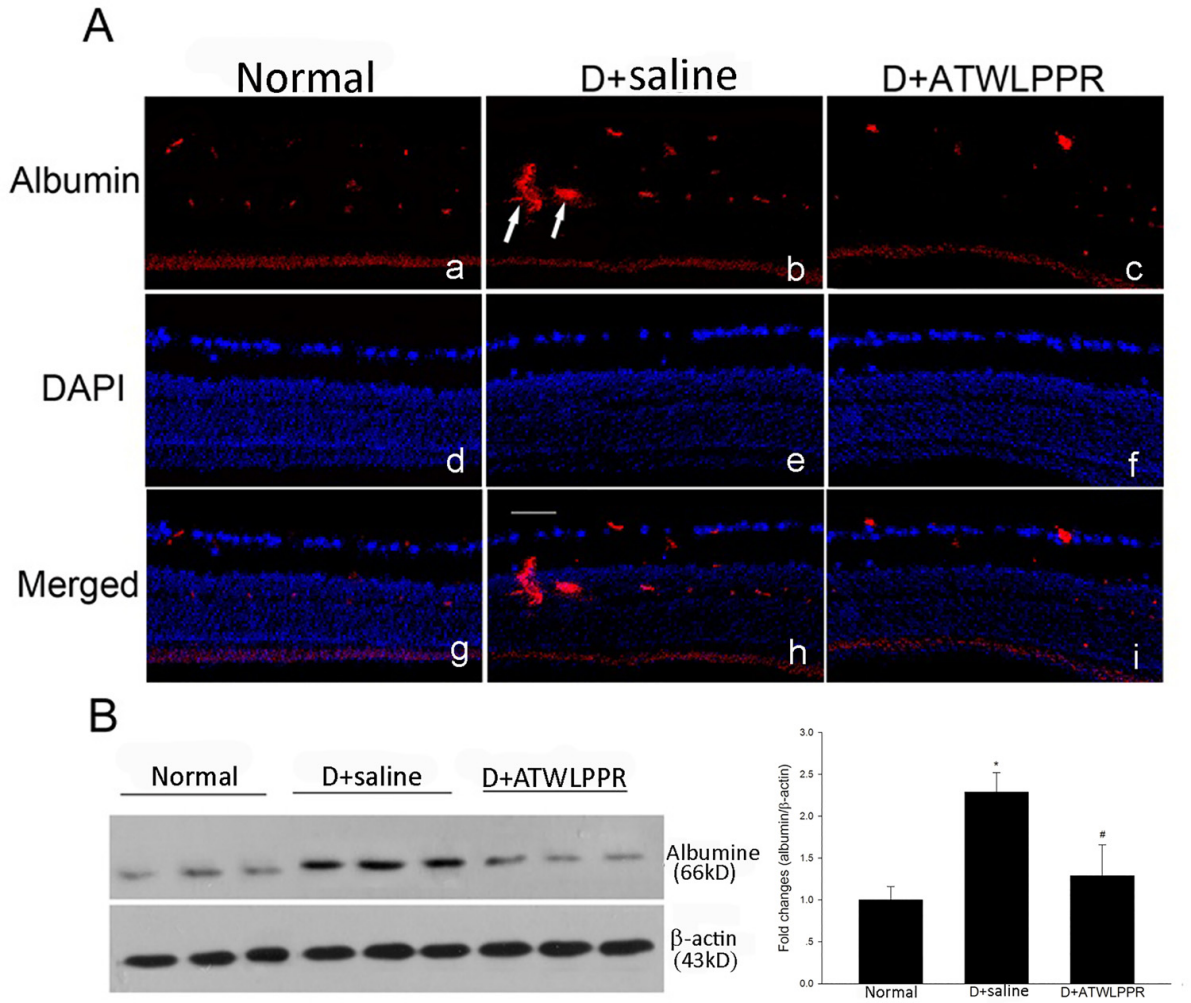
Extravascular albumin was inhibited by ATWLPPR in diabetic retinas. (A) Immunohistochemical staining shows the extravascular albumin (arrows) in retinal sections. Normal (a, d, g); D+saline (b, e, h); D+ATWLPPR (c, f, i). Scale bar = 50 mm. (B) Western blot analysis indicates the significant increase in leaking albumin in the D+saline retinas was suppressed by treatment with ATWLPPR. D, Diabetic. *P < 0.05, versus the Normal group; #*P* < 0.05, versus the D+saline group.

Tight junction proteins are very important for vascular integrity. Western blot analysis showed that the total occludin content was significantly decreased in diabetic mice retina. However, diabetic animals treated with ATWLPPR exhibited a restoration of the occludin content (Fig 5). Albumin can leak out into the parenchyma when the blood-retinal barrier is dysfunctional [2]. Western blot analysis was performed to evaluate the extravascular albumin level. There was a significant increase in the retinal extravascular albumin levels in diabetic mice. This increase was attenuated with ATWLPPR treatment (Fig 6). Under normal conditions, albumin was confined within the retinal vessels where the blood-retinal barrier was intact.

### 3.5 Inhibition of NRP-1 reduced diabetes-induced oxidative stress

**Fig 7:**
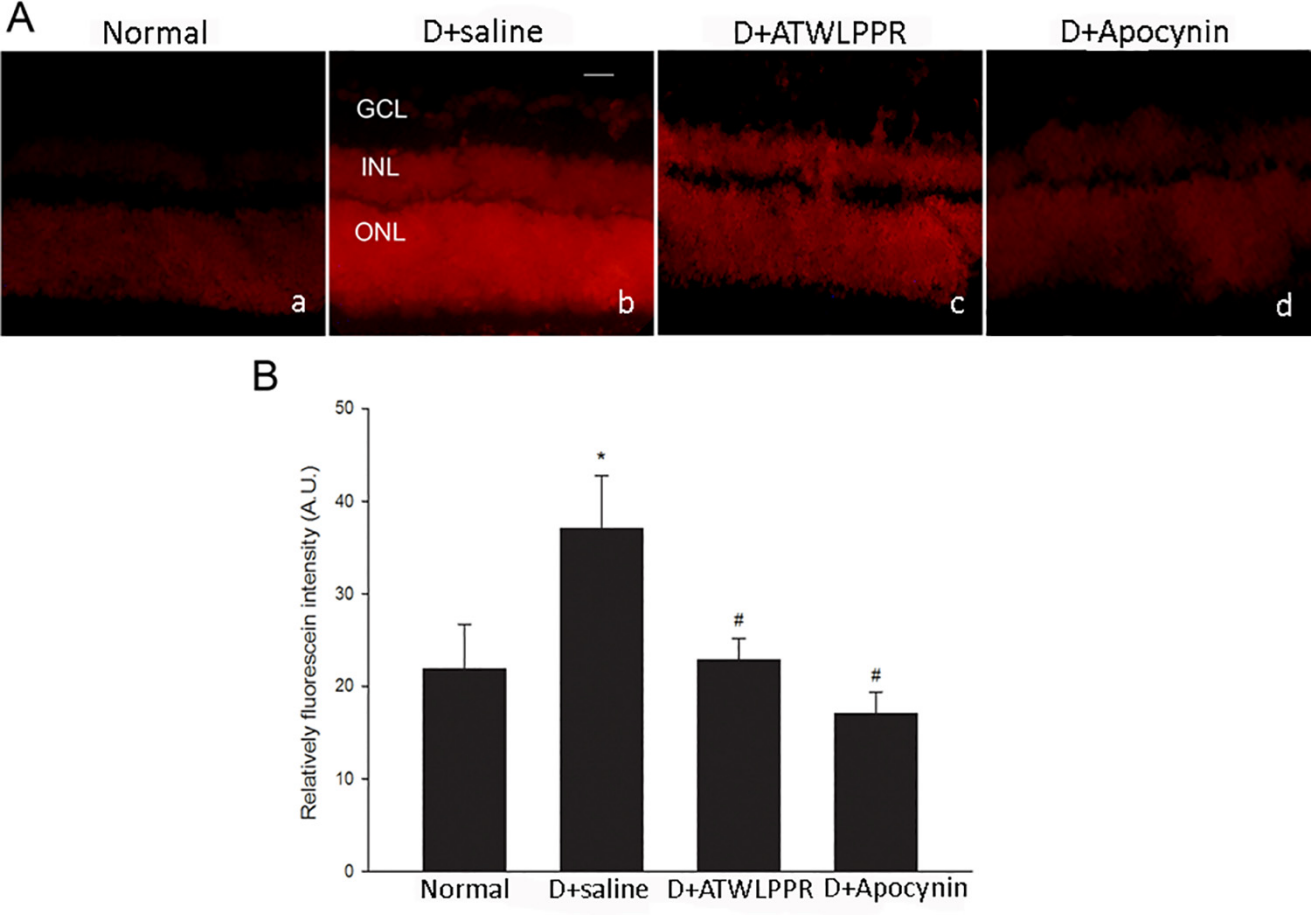
The increase in ROS formation in diabetic retinas was inhibited by treatment with ATELPPR or pretreatment with apocynin (1 mM). (A) Representative images of DHE imaging. (B) Quantitative analysis of fluorescence intensity in DHE images showed an obvious increase in ROS formation in diabetic retinas. ROS generation was significantly reduced in D+ATWLPPR and pretreatment with apocynin (D+apocynin). D, Diabetic. *P < 0.05, versus the Normal group; #*P* < 0.05, versus the D+saline group. Scale bar = 50 mm, 200X. GCL, ganglion cell layer; INL, inner nuclear layer; ONL, outer nuclear layer.

Superoxide production in the retina was assessed using dihydroethidium (DHE) imaging of frozen retinal sections. DHE was oxidized by superoxide to form ethidium after incubating with substrate in snap-frozen sections. Ethidium may bind to DNA and appear as red fluorescence. In the diabetic retina, the DHE-superoxide reaction was increased significantly. The images of ATWLPPR-treated retinas were similar to those of the normal retinas in the DHE reaction. In the control studies, sections from diabetic retinas were pre-incubated with the NADPH oxidase inhibitor, apocynin. The DHE reaction was largely prevented, indicating the specificity of the reaction (Fig 7).

**Fig 8:**
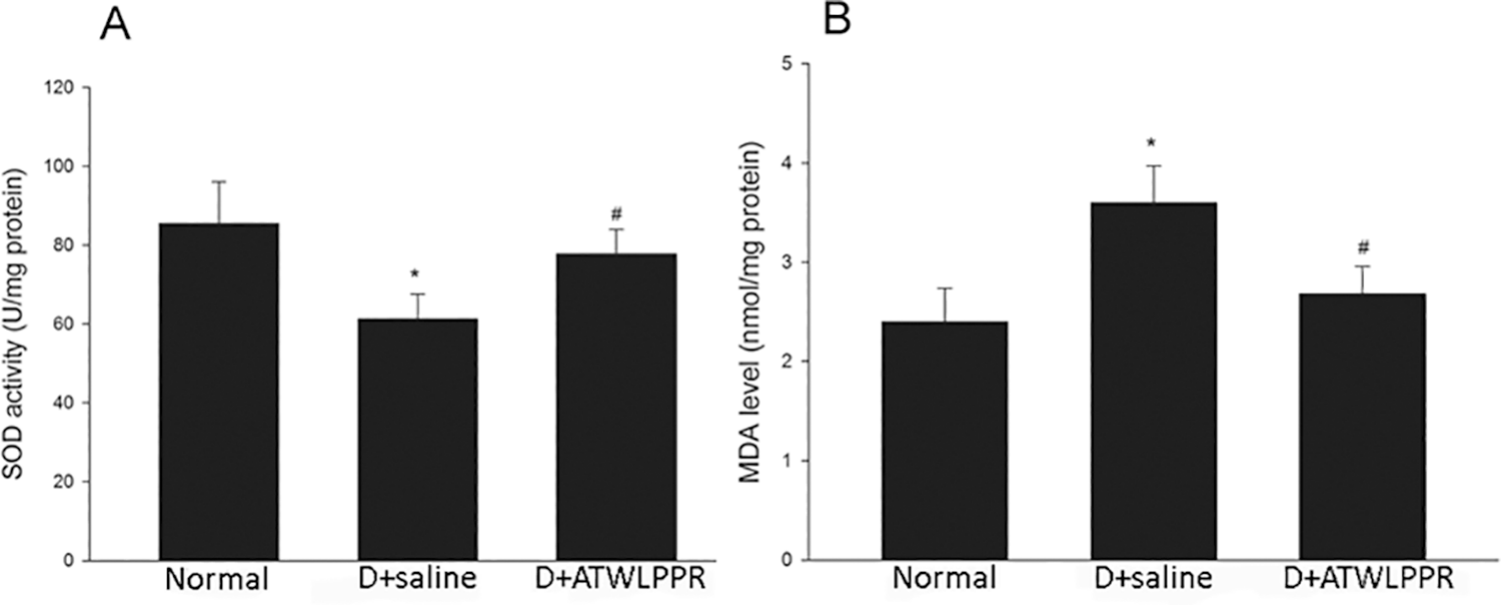
ATWLPPR decreased oxidative stress in the diabetic retinas by regulation of the MDA levels and SOD activity. (A) MDA levels (nmol MDA/mg protein); (B) SOD activity (U/mg protein). D, Diabetic. *P < 0.05, versus the Normal group; #*P* < 0.05, versus the D+saline group.

SOD, an endogenous anti-oxidant enzyme, is also an important free radical scavenger. Another indicator, malondialdehyde (MDA), is a product of lipid peroxidation, which occurs during oxidative stress in tissues or living cells. Both the SOD and MDA levels were determined to evaluate the oxidative response in diabetic retinas and DB+ATWLPPR retinas. The results are shown in Fig 8. In DR retinas, treatment with ATWLPPR significantly increased the activities of SOD and decreased the production of MDA (*P* < 0.05).

### 3.6 The increase in inflammation-associated proteins in the retina was prevented by ATWLPPR in diabetes mice

**Fig 9:**
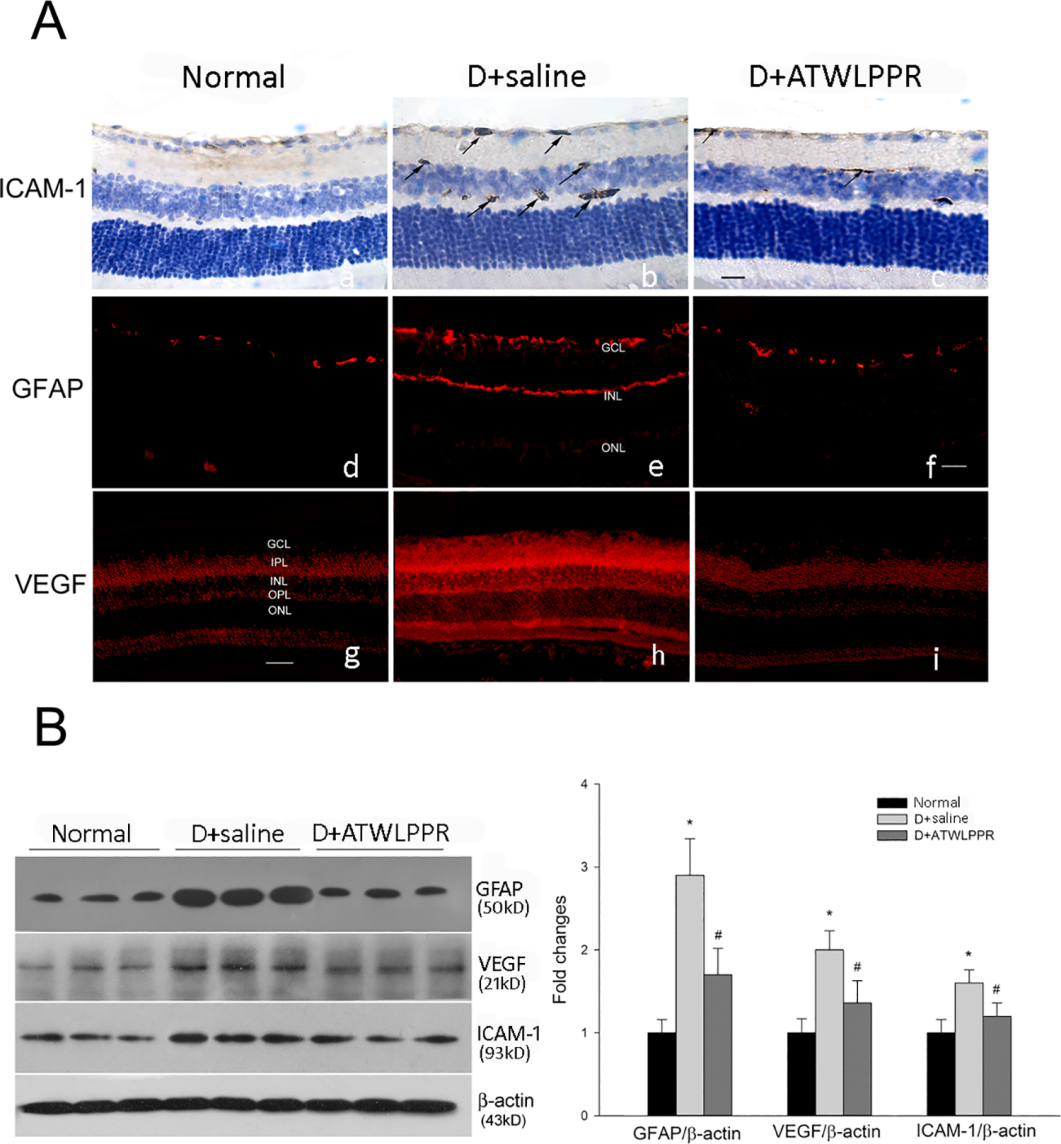
ATWLPPR inhibited the diabetes-induced up-regulation of inflammatory proteins in the retina. (A) Representative immunohistochemical images for ICAM-1 (a, b, c), GFAP (d, e, f), and VEGF (g, h, i) staining on retinal sections. Arrows, immune-positive. Scale Bar = 50 mm, 200X. GCL, ganglion cell layer; IPL, inner plexiform layer; INL, inner nuclear layer; OPL, outer plexiform layer; ONL, outer nuclear layer. (B) Western blot analysis of the GFAP, VEGF and ICAM-1 expression levels in retinas. D, Diabetic. *P < 0.05, versus the Normal group; #*P* < 0.05, versus the D+saline group.

Glial cell activation is a prominent early feature of diabetic retinopathy [17]. The expression of glial fibrillary acidic protein (GFAP) is known to increase in activated glia, which exerts the role of modulation of the inflammatory response. VEGF and ICAM-1 are thought to be crucial proteins that mediate leukostasis and the loss of the blood-retinal barrier properties. Western blot and immunohistochemistry were used to examine the expression of these proteins. Diabetes induced a significant increase in the expression of GFAP, VEGF and ICAM-1, and this increase was markedly attenuated by treatment with ATWLPPR, as shown in Fig 9.

## Discussion

DR is an increasingly common cause of blindness and visual impairment worldwide. The development of DR and its pathogenesis are very complex because of the multiple inter-related mechanisms. VEGF has been considered to be an important factor involved in DR. Anti-VEGF therapy is currently a hallmark strategy for preventing DR. Other than directly targeting VEGF, in this study, we used ATWLPPR peptide, an NRP-1 inhibitor, which was also considered to be a VEGF co-receptor antagonist. The results in the present study indicated that ATWLPPR had retinal protective effects against vascular injury, oxidative stress and up-regulation of GFAP, VEGF and ICAM-1 in a mouse model of the early stages of experimental diabetic retinopathy.

The high levels of blood glucose in diabetic mice were not influenced by treatment with ATWLPPR. In the other words, ATWLPPR did not affect the regulation of blood glucose. The body weight of the diabetic treated mice and diabetic saline mice were significantly different at the 4^th^ and 5^th^ weeks after the induction of diabetes. This result was unexpected. Alessandro Fantin et al. generated Nrp1^Y297A/297A^ mutant mice [18]. They indicated that the expression of NRP1 and the combination of NRP1 with VEGF were decreased in the mutant mice. Furthermore, the body weight of the Nrp1^Y297A/297A^ mutant mice was significantly lower than that of the control mice 6–18 weeks after birth. This result suggested that NRP1, either alone or in combination with VEGF, plays an important role in mouse development. However, a study by Agustin Cerani et al. demonstrated that deletion of NRP1 in the circulatory system (Tam-treated Tg^cre-Esr1^/Nrp^fl/fl^) when mice are 6–8 weeks old had no effects on body weight, size and open-field activity [19]. We thought that the discrepancy with the above results was caused by NRP1 being deleted only in the circulatory system in the later study. This study indicated that the application of the NRP1 inhibitor, ATWLPPR, significantly reduced the body weight gain compared to the diabetic saline group. This was consistent with the work of Alessandro Fantin et al. Additional intensive study is needed to elucidate the detailed mechanism in the future.

In the present study, the ERG findings changed at the end of the fifth week after induced DB. In the DB animals, the b-wave amplitudes were decreased, and the implicit times were increased compared with the normal mice. ATWLPPR treatment abolished the abnormal appearance of the b-waves. Electrophysiological technology detects retinal dysfunction, which usually precedes the neuronal and vascular damage in the preclinical stages of DR [20, 21]. The amplitude and implicit time of the b waves were relatively stable and is usually an indicator in the ERG analysis. Performed several weeks after the induction of DB, the ERG abnormalities have been described in many reports [20–23]. In this study, the first evidence was provided that the application of an NRP-1 inhibitor, ATWLPPR, prevents the b wave abnormalities in the ERG. Retinal microaneurysms are the characteristic hallmark of DR and is also a reliable sign of the early stages of DR [24, 25]. We detected microaneurysms in the retinal fundus of diabetic animals by using FFA technology; however, microaneurysms were not found in ATWLPPR treatment animals, as shown in Fig 3.

In the early stages of DR, vascular permeability dysfunction is an important pathophysiological effect in the progression of DR. The blood-retinal barrier (BRB) disruption may sequentially induce fluid extravasation and leucocyte adhesion and infiltration. Many reports have demonstrated that leukocyte aggregation and infiltration during DR generates a large amount of ROS and inflammatory factors, which aggravate the retinal injury induced by diabetes [26–29]. These findings indicate that vascular integrity is an important factor for the initiation of the pathological processes of DR. Vascular integrity depends on BRB tight junctions which are comprised of occludins, claudins, ZO proteins, and other tight junction-associated proteins [30, 31]. In the present study, we found that STZ injection induced an increase in attached leukocytes and extravasation of albumin and a decrease in occludin expression in the mouse retinas. These changes were observed in the early stages of DR [2, 32, 33]. The application of the NRP1 inhibitor, ATWLPPR, reduced the occludin degradation and preserved the vascular integrity.

As a transmembrane glycoprotein, NRP1 was initially observed in neuronal axons and may elicit repulsive activities after combining with semaphorin 3A [34]. Studies have indicated that NRP1 is also expressed in endothelial cells and functions as a VEGF co-receptor [35]. Co-expression of NRP1 enhances VEGFR2 signaling by up to 6-fold and amplifies the physiological functions including vascular permeability, migration, and proliferation [35]. In contrast, when the blood vessel endothelial cells lack NRP1, the VEGFR2 on the cell surface is internalized and its levels are decreased [36]. This phenomenon also suggests that NRP1 affects the number of VEGFR2 molecules that interact with VEGF and then affects vascular permeability. Sema3a is generated by stressed retinal ganglion cells and is one of the ligands of NRP1. Recently, a study demonstrated that sema3a plays an important role in mediating the breakdown of barrier function in diabetic retinopathy [19]. The other NRP1 ligand, sema3c, has also been most recently shown to act as an anti-angiogenic agent through the inhibition of endothelial junction integrity. Moreover, the application of sema3c effectively inhibited the generation of pathological vessel growth in a ROP model [37]. Therefore, we could not draw a conclusion in the present study that the observed effects are solely due to VEGF inhibition. Sema3a and sema3c were most likely also involved in this pathway through combined action with NRP1.

The breakdown of vascular integrity and leucocyte adhesion and infiltration have been linked to many types of diseases including DR. In this pathological environment, such as DR, the increased generation of ROS induces retinal oxidant stress injury. Previous studies have demonstrated that the MDA level, a marker of lipid peroxidation, was clearly increased and the activity of SOD, an endogenous anti-oxidant enzyme, was significantly decreased in the retinas and serum from DR patients or mice [38–41]. In the current study, the MDA level and SOD activity were assayed in the DR retinas that were treated with and without the NRP1 inhibitor. Superoxide production was also assessed using DHE imaging of frozen retinal sections. All of the results indicated that ROS formation and oxidative stress were significantly increased in the DR retinas, which is consistent with previous reports. Blocking NRP1 using ATWLPPR to reduce the VEGFR2 signaling may decrease the oxidative stress in DR retinas through preservation of vascular integrity. ROS formation and oxidative stress always cause increased expression of pro-inflammatory transcription factors, and the activation of these redox-dependent inflammatory factors may lead to high expression levels of VEGF, ICAM-1 and GFAP [2, 32, 42]. In this study, the results from western blot and immunohistochemistry demonstrated that the increased expression of VEGF, ICAM-1 and GFAP in DR retinas were associated with increased ROS formation. The application of ATWLPPR significantly inhibited these DR induced alternations. Previous reports indicated that the up-regulation of VEGF and ICAM-1 was an early marker of vascular injury in DR [42, 43]. VEGF may promote ICAM-1 expression on vascular endothelial cells [44] and may lead to an increase in vascular permeability [45]. ICAM-1 mediates the circulating leukocytes that come into contact with endothelial cells and induces leukocyte adhesion to the vascular wall [46]. Glial cells or Müller cells surround the vascular and play important roles in retinal microangiopathy and vascular permeability when they are activated by secreted inflammatory factors in DR retinas [47]. In addition to the vascular endothelial cells, pericytes are also an important cell type that associate with capillaries and have been implicated in the maintenance of the blood-brain barrier [48, 49]. Pericytes play an important role in the regulation of angiogenesis and the formation of functional vessels. The loss of pericytes correlates with the neovascularization in early diabetic retinopathy [50]. Therefore, we should pay more attention to the pericytes and should stain pericytes together with endothelial cells on the retina flat mounts in different groups in the future.

In summary, we provide evidence that treatment with ATWLPPR, an NRP inhibitor, in STZ-induced DR retinas preserved the integrity of the vasculature and prevented retinal injury in the early stages of DR through the reduction of oxidative stress and the down-regulation of VEGF, ICAM-1 and GFAP expression. These effects demonstrate that NRP1 plays an important role in mediating VEGFR signaling during the course of DR. Further research is still required to explore the molecular mechanisms of the VEGFR/NRP1 mediated pathway in DR. Taken together, different from other anti-VEGF therapies, this study proposed a possible therapeutic strategy against DR.

## Supporting Information

S1 Datasheet Changes in body weight and blood glucose from the STZ injection. (XLSX)

S2 Datasheet Alternations in amplitude and implicit time of b-wave in ERG recordings. (XLSX)

S3 Datasheet Leukocyte attachment. (XLSX)

S4 Datasheet Western blot analysis the total occludin content. (XLSX)

S5 Datasheet Western blot analysis the leaking albumin. (XLSX)

S6 Datasheet Quantitative analysis the fluorescence intensity in DHE images. (XLSX)

S7 Datasheet The SOD activities and MDA levels. (XLSX)

S8 Datasheet Western blot analysis the expressions of GFAP, ICAM1 and VEGF. (XLSX)
